# Establishing Priority Pediatric Antimicrobial Stewardship Interventions in the US: Findings from a Delphi Consensus Study

**DOI:** 10.3390/antibiotics14101011

**Published:** 2025-10-11

**Authors:** Harry Obeng, Emmanuel Tetteh, Sara Malone, Lauren Walsh, Tyler Walsh, Fernando J. Bula-Rudas, Ritu Banerjee, Adam W. Brothers, Joshua C. Herigon, Katie Namtu, Scott Weissman, Daniel Riggsbee, Jared Olson, Debra Lynn Palazzi, Ann Wirtz, Matthew Sattler, Jessica Tansmore, Brittany A. Rodriguez, Monica Abdelnour, Joshua R. Watson, Alison C. Tribble, Jessica Gillon, Mari Nakamura, Sarah Jones, Jason G. Newland, Virginia R. McKay

**Affiliations:** 1 Brown School, Washington University in St. Louis, St. Louis, MO 63130, USA; h.a.obeng@wustl.edu; 2 School of Public Health, Washington University in St. Louis, St. Louis, MO 63110, USA; emmanuel.tetteh@wustl.edu (E.T.); sara.malone@wustl.edu (S.M.); 3 Division of Pediatric Infectious Diseases, Nationwide Children’s Hospital, Columbus, OH 43205, USA; lauren.walsh3@nationwidechildrens.org (L.W.); tyler.walsh@nationwidechildrens.org (T.W.); jessica.tansmore@nationwidechildrens.org (J.T.); joshua.watson@nationwidechildrens.org (J.R.W.); jason.newland@nationwidechildrens.org (J.G.N.); 4 Department of Medicine, Pediatric Infectious Diseases, Johns Hopkins All Children’s Hospital, Saint Petersburg, FL 33701, USA; fbularu1@jhmi.edu; 5 Pediatric Infectious Diseases, Vanderbilt University Medical Center, Nashville, TN 37232, USA; ritu.banerjee@vumc.org; 6 Department of Pharmacy, Seattle Children’s Hospital, Seattle, WA 98105, USA; adam.brothers@seattlechildrens.org; 7 Department of Pediatrics, The Children’s Mercy Hospital, School of Medicine, University of Missouri-Kansas City, Kansas City, MO 64108, USA; jherigon2@cmh.edu; 8 Infectious Disease Clinical Pharmacy, Johns Hopkins All Children’s Hospital, Saint Petersburg, FL 33701, USA; katie.namtu@jhmi.edu; 9 Pediatric Infectious Diseases, Seattle Children’s Hospital, Seattle, WA 98105, USA; scott.weissman@seattlechildrens.org; 10 Department of Pediatrics, Division of Pediatric Infectious Diseases, C.S. Mott Children’s Hospital, University of Michigan, Ann Arbor, MI 48109, USA; dariggsb@med.umich.edu (D.R.); tribblea@med.umich.edu (A.C.T.); 11 Pediatric Infectious Diseases, University of Utah, Salt Lake City, UT 84113, USA; jared.olson@imail.org; 12 Pediatric Infectious Diseases, Texas Children’s Hospital, Houston, TX 77030, USA; dpalazzi@bcm.edu; 13 Department of Pediatrics, Baylor College of Medicine, Houston, TX 77030, USA; 14 Department of Pharmacy, Children’s Mercy Kansas City, Kansas City, MO 64108, USA; alwirtz@cmh.edu; 15 School of Pharmacy, University of Missouri–Kansas City, Kansas City, MO 64108, USA; 16 Department of Pediatrics, Division of Infectious Diseases, Washington University in St. Louis School of Medicine, St. Louis, MO 63110, USAmabdelnour@wustl.edu (M.A.); 17 Department of Pharmacy, Texas Children’s Hospital, Houston, TX 77030, USA; barodri1@texaschildrens.org; 18 Department of Pharmacy, Monroe Carell Jr Children’s Hospital, Nashville, TN 37232, USA; jessica.gillon@vumc.org; 19 Antimicrobial Stewardship Program and Division of Infectious Diseases, Boston Children’s Hospital, Boston, MA 02115, USA; mari.nakamura@childrens.harvard.edu (M.N.); sarah.jones@childrens.harvard.edu (S.J.); 20 Department of Pediatrics, Ohio State University College of Medicine, Columbus, OH 43210, USA

**Keywords:** intervention, stewardship, metrics, antibiotics, antimicrobials

## Abstract

Background/Objectives: Antimicrobial resistance (AMR) is a major global health threat, with children at higher risk due to developmental differences in drug metabolism, limited treatment options and inappropriate antibiotic use. Pediatric antimicrobial stewardship programs (ASPs) face implementation challenges, often relying on adult-based guidelines and limited pediatric-specific evidence. This study aimed to identify and prioritize the most critical areas for pediatric ASP intervention development through a structured, multi-round Delphi consensus process with experts in antimicrobial stewardship and infectious diseases. Method: A four-round modified Delphi process was conducted to identify and prioritize key pediatric ASP interventions. Experts in antimicrobial stewardship and infectious diseases were recruited through an existing clinical trial. Using an iterative survey and in-person discussions, experts provided input on priority areas, which were thematically grouped and refined across rounds. Structured feedback supported real-time refinement and consensus-building. Results: Twenty experts participated in the process, generating 25 priority items in Round 1 through open-ended responses. These were narrowed to seven key priorities through structured voting and discussion. The final items were clustered into three intersecting themes: Care Settings, Prescriptions, and Strategies. Care Settings focused on high-impact areas such as outpatient clinics and intensive care units, where misuse is common and/or care is complex. The prescriptions theme prioritized shorter durations and narrow-spectrum agents. The strategy theme highlighted the need for outcome-based metrics, improved diagnostic stewardship, and routine tracking of patient outcomes to guide and assess stewardship efforts. Conclusions: This expert consensus identified key priorities for pediatric ASPs, providing a foundation for future interventions. Findings can be used to inform policy and practice, improving the appropriate use of antimicrobials in pediatrics and combating AMR.

## 1. Introduction

Antimicrobial resistance (AMR) is a global public health emergency. It currently ranks among the top ten global health threats identified by the World Health Organization (WHO) [1]. Recent estimates indicate that drug-resistant infections cause almost 5 million deaths yearly [1]. This figure is expected to rise to 10 million deaths per year by 2050, which could cost the global economy over $100 trillion if left unchecked [2,3]. Antimicrobial stewardship initiatives are now crucial for encouraging the prudent use of antimicrobials to maintain their effectiveness [4].

The implementation of antimicrobial stewardship strategies to curb AMR, however, remains challenging, especially in pediatric settings, where particular clinical and physiological variables add complexity relative to adult settings. AMR poses a significant threat to children because of factors such as unnecessary medication use, physiological differences in drug metabolism, and fewer treatment options [2]. In 2016, antimicrobial resistance was estimated to cause around 214,000 deaths in newborns worldwide, and by 2019, about one-fifth of all bacterial AMR-related fatalities occurred in children under five years old [1]. In 2022, more than 3 million pediatric deaths globally were attributed to infections associated with AMR [5]. Notably, an estimated 2 million of these deaths were linked to the use of higher risk ‘Watch’ and ‘Reserve’ antibiotics rather than first line drugs, underscoring the urgent need for improved antimicrobial stewardship in pediatric care [5]. Overuse and underuse of antimicrobials jeopardize the management of potentially life-threatening pediatric infections by accelerating the development of resistance [6]. Additionally, most current antimicrobial stewardship guidelines are derived from adult data, regardless of notable variations in pediatric drug pharmacokinetics and resistance patterns, which exacerbate these difficulties [7].

Yet, opportunities abound to reduce excessive antibiotic use in pediatrics. Estimates suggest that as many as half of the antibiotic prescriptions given to pediatric patients are either redundant or inappropriate. This is substantially greater compared to that of adult patients [8]. According to McMullan et al., surgical prophylaxis antibiotics were deemed to be inappropriate in 59% of cases among hospitalized children in Australia [9]. This rate is notably higher than the 42.6% rate of inappropriate antimicrobial prescribing reported across the same National Antimicrobial Prescribing Survey (NAPS) cohort from 2014 to 2017, reflecting all prescription types in hospitalized children [10]. With increasing interest in hospital antimicrobial stewardship programs (ASPs), core elements of ASPs have been defined based on principles of effectiveness and affordability [10]. ASPs are then charged with supporting the establishment of guidelines and education for health providers [11]. A Cochrane review conducted by Davey et al. suggests that interventions aimed at enhancing compliance with policies regarding the use of antibiotics in hospitals are mostly successful, especially in minimizing treatment length [12]. However, the authors suggested bringing together experts in antibiotic stewardship with social scientists to form a coherent strategy that directs both research and ultimately practice towards the better-controlled development of hospital-associated ASPs [12].

Expert consensus is essential in forming antimicrobial stewardship-focused policies and procedures. The modified Delphi method serves as an organized, iterative procedure for reaching expert consensus on complicated matters allowing variable anonymity and facilitating interaction among panelists [13,14]. It is especially helpful in healthcare studies when there is a lack of direct evidence for establishing priorities, creating guidelines for care or determining the most effective approaches. For example, the Delphi technique has been used recently in antimicrobial stewardship to develop quality indicators for the use of antibiotics in emergency rooms and to create best practice guidelines for peer comparison and audit and feedback [15,16]. This study aimed to systematically identify and prioritize the most critical, actionable interventions for improving antimicrobial stewardship in pediatric healthcare settings. By engaging multidisciplinary experts, we aimed to establish an evidence-based hierarchy of stewardship priorities to guide clinical practice and future research in pediatric antimicrobial use. Ultimately, this effort aims to guide the development of institutional and public policies, inform the allocation of resources, and strengthen both the implementation of antimicrobial stewardship strategies and the pediatric research agenda.

## 2. Results

A total of 20 participants were recruited for the study. Most participants were either an Infectious Disease physician or pharmacist. The majority of participants identified as white, and gender distribution was relatively even. Participant demographics are described in detail in Table 1.

### 2.1. Round 1—Initial Idea Generation

A total of 17 of 20 (85%) participants completed the survey. The reason for the three experts’ non-participation is not being present for this round. Of the 19 respondents with demographic data, half were infectious disease physicians, and 40% were pharmacists. Most reported 11–20 years of professional experience (50%), with 8 (40%) reporting 5–10 years and 1 (5%) more than 20 years. A total of 41 priority items were gathered from participants. The voting responses were categorized into 3 main themes as shown in Table 2. The Strategies theme was further divided into Clinical Practice Strategies and Programmatic and System-Level Strategies due to the large number of variations. Care Settings captured the various healthcare environments in which antimicrobial stewardship efforts are implemented. Prescriptions related to the foundational elements that define how antimicrobials are prescribed. The Strategies theme generally captured clinical practice strategies and pragmatic and system-level strategies. Clinical Practice Strategies were initiatives that have a direct impact on daily clinical prescribing and decision-making. In contrast, Programmatic and System-Level Strategies were more comprehensive organizational, structural, and evaluative methods that help to maintain and promote good antimicrobial stewardship practices. When results were presented back to participants for refinement, key adjustments included the recategorization of some items and a suggestion to introduce an extra item after initial data collection. For example, inpatient antimicrobial stewardship was added to Care Settings. Finally, efforts focused on continued surgical prophylaxis, patient care outcomes, and the antibiotic harm index were incorporated into Programmatic and System-Level Strategies.

### 2.2. Round 2

This round aimed to reduce the number of items identified in the previous phase and focus on consolidating the most critical aspects of ASP. Participants were encouraged to discuss the results and then vote on priorities. Various categories were explained in further detail to provide more context for participants before voting.

All 20 recruited (100%) participants completed the prioritization survey. Table 3 presents the distribution of votes for the various selections under each theme. Participants could select two priorities per theme per round. Thus, the number of votes represents the total number of selections and may exceed the total number of individuals. The highest-ranked item overall was duration of therapy (17 votes, 85% consensus) within the Prescriptions theme, with participants noting that durations are often arbitrary and a major driver of antibiotic overuse. Another highlighted item in the Prescriptions theme was narrow versus broad spectrum selection for community-acquired infections (5 votes, 25% consensus). There was broad agreement that optimizing duration is a high-impact, actionable target. Outpatient stewardship (13 votes, 65% consensus) emerged as the highest-ranking item in the Care Settings category. Participants emphasized that despite being the site of most antibiotic prescribing, antimicrobial stewardship efforts here remain limited, with high patient volumes, limited oversight, and many prescriptions potentially being unnecessary. Antimicrobial stewardship in the intensive care unit (ICU; 6 votes, 30% consensus) was also highlighted because these settings have high complexity as well as extensive antibiotic use and, not infrequently, resistance to antimicrobial stewardship recommendations. Within Programmatic and System-Level Strategies, metrics for monitoring outcomes (10 votes, 50% consensus) was the highest ranked item. Many participants expressed concern that current metrics focus too heavily on process measures rather than clinical outcomes; calling for data that better demonstrates the impact of ASPs on patient care and outcomes. One significant decision in this round for this theme was merging an antibiotic harm index and granular measures (for example—the proportion of surgical prophylaxis cases with antibiotic discontinuation within 24 hours—metrics that enable precise tracking of guideline adherence and prescribing behavior) with the broader metrics category. The consensus was that by consolidating measures, ASPs could concentrate on fewer but more meaningful indicators of success. From the perspective of the participants, this restructuring would enable a more robust evaluation of ASP impact and facilitate the development of clear and actionable performance indicators. After dropping 17 items (2 from Care Settings, 3 from Prescriptions, 7 from Clinical Practice Strategies, and 5 from Programmatic and System-Level Strategies), high-priority items from Clinical Practice Strategies and Programmatic and System-Level Strategies were then consolidated for voting to identify the most critical focus areas for antimicrobial stewardship.

### 2.3. Round 3

In the third round of the Delphi process, 17 participants focused on voting for the highest-priority selections from the Strategies (Theme 3) to determine which strategies should advance to the final consensus round. Items that received two or fewer votes were dropped from further consideration. Meanwhile, the selected priorities in the Care Settings and Prescriptions categories were maintained for further deliberation in Round 4.

The top selections at the end of the round were metrics (13 votes, 76% consensus), followed by diagnostic stewardship and patient outcome tracking, each receiving 2 votes (12% consensus). Participants again emphasized the importance of shifting from traditional process-based evaluations to outcome-driven measurements, which offer a more meaningful gauge of the efficacy of antimicrobial stewardship. Some participants voiced their concern that the actual effects of antimicrobial stewardship actions are not adequately captured by the current data collection techniques. Therefore, there was a broad consensus that the main goal going forward should be to develop standardized, clinically useful stewardship metrics. Many participants also emphasized that by ensuring that diagnostic procedures support appropriate prescribing, diagnostic stewardship has the potential to enhance antibiotic decision-making.

**Figure 1:**
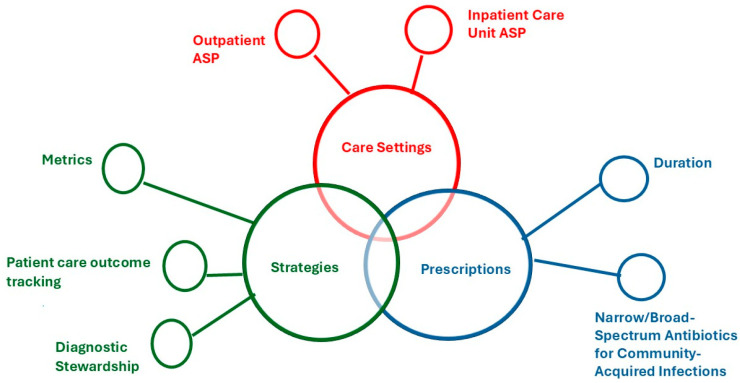
Final Consensus- Based Priorities.

Similar to metrics the patient care outcomes item emphasized how crucial it was to the experts that the direct clinical impact of ASP be evaluated, rather than depending only on procedural compliance or prescribing trends. Central to this item, participants felt that adding patient-centered measures to ASP assessments could improve the overall evaluation of program efficacy. Results from this round are presented in Table 4 and Figure 1.

Figure 1 presents the final priority items for antimicrobial stewardship (ASP), categorized into three overlapping domains: Care Setting, Prescriptions, and Strategies. Each theme includes specific focus areas identified during the prioritization process. Key items under each theme are depicted as nodes branching from the central themes.

### 2.4. Round 4

Discussion in Round 4 provided a refined focus on ASP priorities, particularly in ICU and outpatient settings. Each priority area was discussed with experts to elaborate on its importance and the reasons it is considered essential. Experts discussed the importance of antibiotic duration, safety considerations, and diagnostic accuracy in optimizing antimicrobial use. Many of the items identified were interrelated within and across themes. The consensus points highlighted the need for evidence-based guidelines, robust metrics, and a stronger emphasis on appropriate prescribing to enhance patient outcomes and reduce the risks associated with antibiotic misuse. Supporting evidence for each priority item can be found in Table 5.

#### 2.4.1. Care Settings

##### ASP in the ICU

Experts identified several opportunities to strengthen antimicrobial stewardship efforts specifically within the ICU context. One major concern identified was the unnecessary or inappropriate collection of cultures, which can lead to the identification of contaminants or colonizing organisms and result in misinformed treatment decisions. Experts emphasized the importance of carefully assessing prolonged, ineffective treatments, suggesting that antibiotic extension decisions should be guided by an antibiotic harm index. Additionally, stopping antibiotic therapy in cases of diagnostic uncertainty was highlighted as a crucial practice to prevent unnecessary antibiotic exposure and potential harm (Table 5, Quote 1.1, 1.2).

##### Outpatient ASP

Outpatient settings were also recognized as high-impact intervention areas, especially due to the volume of prescriptions and the lack of consistent guidelines. Experts cited limited awareness of current evidence, as well as the absence of diagnostic support and time constraints, as key challenges. They advocated for improved prescriber education and tools that support accurate, appropriate prescribing in the outpatient environment (Table 5, Quote 1.3). The group emphasized that outpatient ASPs would require innovative and scalable approaches, since traditional inpatient strategies may not be feasible in these decentralized environments.

#### 2.4.2. Prescriptions

##### Duration

The discussion on antibiotic duration reinforced the principle that shorter courses are generally preferred, although widely accepted optimal durations remain undefined for many conditions and effectiveness studies are needed to establish guidelines. In the outpatient setting, a lack of standardized guidelines and limited awareness of recent research contribute to inappropriate prescribing. A recurring issue identified was the unnecessary continuation of antimicrobials in outpatient settings following prolonged inpatient courses (Table 5, Quote 1.4). Experts emphasized the need for both clinical documentation and stewardship guidelines to include specific antibiotic durations rather than broad ranges, to promote clarity and consistency in treatment plans (Table 5, Quote 1.5). Furthermore, participants highlighted the burden antibiotic regimens can place on families, particularly with complex dosing schedules—emphasizing the potential value of simplifying treatment plans to improve adherence and reduce stress. Calls for additional studies validating the efficacy of shorter treatment durations were made, alongside recommendations to track and address antibiotic underuse as part of optimization efforts.

##### Narrow/Broad-Spectrum Antibiotics for Community-Acquired Infections

The experts mentioned that challenges in selecting appropriate-spectrum antibiotics remain a significant concern, particularly in outpatient settings where prescribing often defaults to broad-spectrum agents despite narrow-spectrum agents being appropriate. This underscores two interrelated issues: the persistence of unnecessarily long treatment durations and the inappropriate selection of broad-spectrum antibiotics when narrower options would suffice. These problems are exacerbated by gaps in discharge planning and limited access to current guidelines in high-volume outpatient environments. Experts also highlighted the importance of demonstrating that unnecessarily long antibiotic courses and inappropriate use of broad-spectrum agents can be harmful (Table 5, Quote 1.6). Participants called for improved alignment between treatment decisions and current evidence, particularly through clearer prescribing guidance, better discharge protocols, and education tailored to urgent and primary care providers.

#### 2.4.3. Strategies

##### Metrics

Actionable metrics to monitor outcomes were identified as a priority, with experts emphasizing the need for more insightful, structured approaches to antimicrobial stewardship that would allow a better understanding of how ASPs were functioning and comparison nationally. Metrics were classified into three primary categories: process measures, outcome measures, and program metrics, which would facilitate comparisons across antimicrobial stewardship programs. Additionally, experts called for the development of utilization standards that go beyond DOT, advocating for appropriateness as the gold standard for measuring antibiotic use (Table 5, Quotes 1.7).

The discussions also highlighted the role for a measure like an antibiotic harm index in assessing patient safety that would help balance the risk and benefit of specific antibiotics. A key question raised was how to better integrate patient safety considerations into antimicrobial stewardship strategies. Experts stressed that antibiotic use should be justified by clear patient benefits; otherwise, it presents a risk rather than a therapeutic advantage (Table 5, Quote 1.8).

##### Patient Care Outcome Tracking

Experts consistently stressed that antimicrobial use must be justified by clear patient benefits in contrast to the frequent practice of using antibiotics as a precautionary action. Without demonstrated benefit, antimicrobials present an excess risk of harm. The conversation focused on better integrating patient safety considerations into antimicrobial stewardship strategies. The call to move beyond procedural compliance and toward patient-centered metrics highlights a shift toward outcome-based ASP evaluation, reinforcing the clinical relevance and impact of antimicrobial stewardship efforts (Table 5, Quote 1.9).

##### Diagnostic Stewardship

The issue of unnecessary diagnostic testing, often contributing to increased healthcare costs without improving patient outcomes, was a key focus of the discussion on diagnostic stewardship. Experts emphasized that the overuse of diagnostic tests not only drives substantial economic costs but also poses clinical risks, such as false positives, patient anxiety, and cascades of unnecessary treatment (Table 5, Quote 2.0). They called for more detailed data on how diagnostic tests are being used, including indications, frequency, and clinical context, to uncover patterns of misuse and guide efforts to minimize unnecessary testing.

## 3. Discussion

The priorities identified through this Delphi process represent key directions for the next phase of pediatric antimicrobial stewardship efforts, particularly in areas where further development and research are needed. These findings are intended to inform future research, resource allocation, and programmatic innovation, complementing rather than replacing existing stewardship infrastructure. Through our Delphi process, pediatric antimicrobial stewardship experts reached a consensus on key priorities, focusing on outpatient and ICU antimicrobial stewardship, prescriptions, and strategic approaches to optimizing antimicrobial use. The discussions centered on critical areas, including the duration of antibiotic use, the appropriate selection of narrow- or broad-spectrum antibiotics for community-acquired infections, the development of actionable metrics, diagnostic stewardship, and improving patient outcomes. We provide some specific examples and opportunities in each of the areas that were highlighted. However, there was diversity in opinion about priorities, and no singular item achieved unanimous consensus suggesting multiple areas of potential value for research and stewardship practice. Finally, there are still many circumstances for which there are still not evidence-based guidelines for appropriate prescribing in pediatrics which highlights a general need for additional clinically focused research in addition to the stewardship needs emphasized here.

### 3.1. Strategies—Meaningful Metrics and Patient Outcomes

A clearly articulated priority was the creation and implementation of actionable, outcome-focused metrics. It was pointed out by the participants that conventional measures like days of therapy (DOT) seldom capture the clinical meaning or safety of many medications. This is consistent with emerging calls in the literature to shift evaluation frameworks for ASP toward clinical outcomes, appropriateness of care, and measures of patient harm [17,18]. Recent pediatric studies on innovative stewardship metrics such as linking prescribing diversity or variation to clinical outcomes highlight the ongoing move away from simple volume-based measures toward more clinically meaningful indicators [19]. These opinions are in line with Moehring et al., who pointed out that clinical results and appropriateness are the most important factors for ASP evaluation, surpassing volume-based tracking [20]. The ASP needs to, for instance, sharpen care metrics in the outpatient setting, which is included in an overall antibiotic stewardship plan. Clinical outcome measures are likely more meaningful than cost or process metrics, as they reflect improvements in patient care. However, they are not well-defined, and many antimicrobial stewardship experts are hesitant to measure them routinely [21]. Although it can be difficult to attribute improvements directly to ASP interventions, clinical outcome metrics should be further developed, and standardized, particularly for benchmarking purposes [21]. Evidence from studies showing that ASP interventions can improve patient outcomes provides a foundation for programs to prioritize and refine patient-centered measures. 

Outpatient settings emerged as a key setting for improved stewardship metrics. Outpatient clinics are responsible for more than 60% prescribed antibiotics and use unnecessarily broad-spectrum agents in place of first-line antibiotics almost half the time [21]. Currently, there are no standard metrics for outpatient ASP [21]. Similarly, for the inpatient setting, although there have been a number of possible metrics put forward for hospital ASPs, very few of them have been sufficiently tested to justify inclusion in ongoing program evaluations [20]. In the outpatient context, scoping reviews and consensus studies highlight that the wide variation in existing metrics limits benchmarking and emphasize the need for simple, validated indicators linked to diagnosis and appropriateness, reinforcing our call to establish standardized outpatient ASP measures [22]. Our consensus also echoes earlier pediatric priority-setting efforts that used a multi-stakeholder Delphi in pediatric healthcare-associated infections and antimicrobial research to identify patient-level harm index (metrics) as a high-impact target for pediatric AMR research [23].

### 3.2. Diagnostic Stewardship

Participants acknowledged the value of improved test interpretation and more appropriate utilization in minimizing unnecessary prescribing. Nevertheless, there are still barriers to the implementation of diagnostic stewardship, particularly in pediatrics, where the potential for serious infections drives the use of broad-spectrum antibiotics. This pattern was echoed by Messacar et al. concerning age-tailored, algorithm-supported diagnostics and age-specific decision-making [24]. The goal of antimicrobial stewardship for rapid diagnostic implementation is the timely and correct interpretation of the result to facilitate appropriate diagnosis, treatment, and optimal outcomes while minimizing unnecessary antimicrobial use [24]. Sick-Samuels and Woods-Hill (2022) also highlighted the need to adopt multifaceted, interdisciplinary approaches to optimize both diagnostic processes and antimicrobial use in critically ill children [25]. By limiting unnecessary testing in patients with a low pre-test probability of infection, diagnostic stewardship can, in turn, reduce unwarranted antibiotic exposure [25]. Several studies have examined how diagnostic stewardship interventions influence blood culture ordering, for example, though few have directly assessed their impact on antimicrobial use [26]. In a pediatric ICU, introducing a checklist and decision algorithm for blood culture orders led to a 46% decrease in cultures without adverse effects on mortality or readmissions but impact on antibiotic usage was unclear [26]. Recent work by Claeys & Johnson has stressed the need to incorporate novel diagnostic stewardship interventions in ASPs [26]. Close collaboration with laboratories is required to support diagnostic and antimicrobial stewardship. Diagnostic stewardship faces several challenges, however, including variability in laboratory practices, lack of standardized testing criteria, and inconsistent reference ranges that may limit clinical utility [26]. Research on successful diagnostic stewardship implementation could provide needed guidance broadly.

### 3.3. Prescribing Targets—Treatment Duration and Community-Acquired Infections

There was wide agreement that more evidence is needed to appropriately reduce antibiotic duration to the fewest number of doses or days and select appropriately narrow or broad antibiotics for community-acquired infections. The principle that shorter durations can be as effective as longer durations and lessen the chances of resistance, adverse effects, and costs of patient care, but there are very few guidelines to support treatment regiments. In the specific instance of pediatric community-acquired pneumonia (CAP), previous studies showed superiority of 5-day vs. 10-day courses for CAP [27]. This recommendation aligns with evidence from a comprehensive meta-analysis of 16 randomized trials involving 12,774 children, which demonstrated that short courses (≤5 days) were as effective as longer regimens in terms of clinical cure, treatment failure, relapse, mortality, hospitalization, and adverse events [28]. ASPs and healthcare personnel should prioritize promoting shorter-duration antibiotic courses for children with uncomplicated CAP who are managed as outpatients and treated with oral antibiotics [27,28]. Evidence from a U.S. observational cohort study indicates that short-course therapy (median 6 days) does not increase the odds of 30-day treatment failure compared with longer regimens (median 10 days) [29]. These findings, together with randomized trial data, reinforce the panel’s recommendation that short-course therapy is a safe, effective, and stewardship-aligned strategy to reduce unnecessary antibiotic exposure and limit antimicrobial resistance. Given the high incidence of CAP-related hospitalizations and antibiotic prescriptions in children, reducing treatment duration could have a significant public health impact by limiting unnecessary antibiotic exposure and associated adverse events. Yet, it is unclear how often these recommentations are implemented in practice. Recognizing that evidence determining optimal treatment durations for many conditions may be limited, our experts, in alignment with other recommendations, emphasized a strong need for dedicated clinical trials and implementation studies designed for pediatrics with the focus to establish treatment duration guidelines that are feasible and practical [30].

### 3.4. Care Settings—ICU and Outpatient ASP

Both the outpatient and ICU settings were flagged as high-priority settings for antimicrobial stewardship interventions. ICU care is complicated by the necessity of prompt empiric therapy amidst frequent diagnostic uncertainty. Experts argued that there is infrequent re-evaluation of culture results and treatment duration, and they stressed the need for metric measures like the antibiotic harm index to make decisions about changes to therapy [31]. On the other hand, outpatient antimicrobial stewardship faces challenges like inadequate supervision, lack of on-site diagnostics, and limited provider time with each patient [18,32]. Regardless, the outpatient realm provides a great opportunity for intervention due to the high volume of antibiotic prescriptions. Effective outpatient stewardship relies on strong leadership, ideally through a designated clinician with expertise in antibiotic use, supported by organizational commitment—including administrative backing, salary support, and authority to drive change [33]. ICU and outpatient antimicrobial stewardship programs can reduce unnecessary antibiotic use by systematically following up on negative culture results and discontinuing empiric therapy. Key challenges include establishing reliable discontinuation processes and ensuring effective clinician–patient communication, which may be supported through targeted communication training [33]. Didactic methods such as structured educational sessions on prescribing guidelines combined with clinical decision support and audit-feedback systems have reduced antibiotic prescribing in outpatient settings, especially for children [34]. Studies emphasize that the social and behavioral context of clinical encounters plays a significant role in shaping antibiotic prescribing in outpatient settings [33]. Moreover, educating caregivers about the typical progression of common infectious can help align expectations and decrease unnecessary demand for antibiotics.

### 3.5. Limitations

There are several limitations to this study. First, the expert panel was primarily recruited from participants in the OPerAtiC trial and affiliated academic institutions, which may limit the generalizability of the results to community hospitals or low-resource settings where antibiotic prescribing patterns and stewardship challenges may differ. However, we achieved a critical mass of individuals who are suitable for Delphi designs, ensuring good representation. Second, while the first round of the Delphi process was anonymous, subsequent rounds involved open discussions, which could have introduced social desirability bias, with participants potentially hesitant to dissent from emerging consensus views. This may have led to an overemphasis on certain priorities, such as ICU stewardship, at the expense of other important areas, but in our observation, there was broad engagement by most participants, expressing diverse opinions. Third, the study did not evaluate the cost-effectiveness or practicality of the suggested interventions, thus certain suggestions might be challenging to put into practice in hospitals with little funding. Future studies should assess the practicality of these stewardship techniques and incorporate a wider range of healthcare environments.

## 4. Methods

### 4.1. Overview

**Figure 2:**
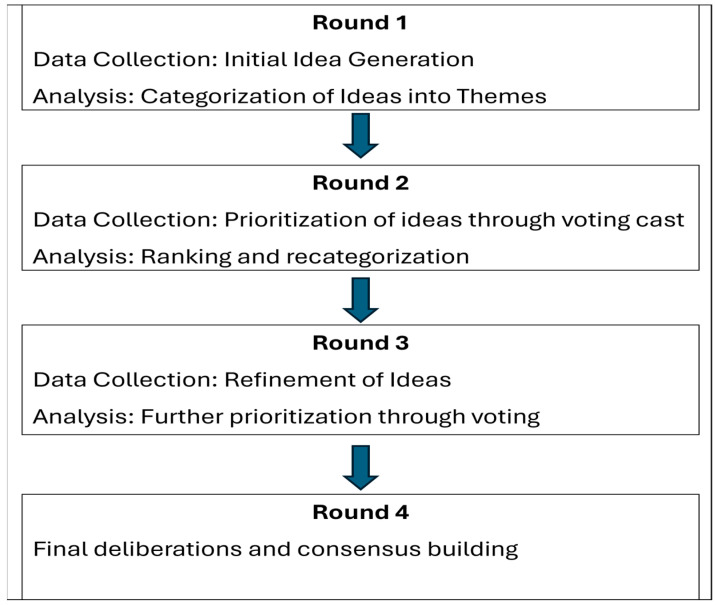
Delphi Process Flow Diagram: From Idea Generation to Consensus.

This study employed a modified Delphi process to reach expert consensus on priority areas for ASP intervention in pediatric care. The Delphi method is a structured research technique that achieves consensus among a panel of experts through iterative rounds of surveys and feedback [16]. The study followed a four-round approach, with participants reviewing, rating, and refining ASP priorities at each stage. In Round 1, participants provided initial, independent input. Then, in Rounds 2 and 3, key themes from the previous round were presented on categorized boards during in-person prioritization exercises; individually, participants wrote down their top priorities and displayed them on corresponding theme boards. Round 4 consisted of facilitated verbal discussions, allowing participants to elaborate on their selections and collaboratively refine the final priorities. After each round of prioritization, we invited participants to discuss why they selected their items. Figure 2 (Delphi Process Flow Diagram: From Idea Generation to Consensus) illustrates the process over the rounds. The process was designed to be dynamic, allowing participants to adjust their responses based on group feedback, which is facilitated by a real-time digital platform. The study protocol was reviewed and approved by the Institutional Review Board at Washington University in St. Louis and was determined to be an exempt quality improvement study. Participants were not given an incentive for participation.

### 4.2. Recruitment of Participants

Participants were purposively sampled from US pediatric hospitals participating in the Optimizing Perioperative Antibiotic in Children (OPerAtic) trial [35], as well as from local institutions, to ensure broad representation. We specifically engaged experts in antimicrobial stewardship, infectious diseases, and pharmacy. Inclusion criteria required at least five years of relevant professional experience, demonstrated expertise through publications or leadership roles, and familiarity with antimicrobial stewardship strategies in pediatric and adult populations. Recruitment was conducted via email invitations to participants, and as part of the recruitment, participants were given a description of the study. We aimed to recruit a minimum of 20 experts based on existing studies, which suggest expert consensus and saturation can be reached with 11–50 participants [36,37]. The study was conducted from 24 to 26 February 2025.

### 4.3. Data Collection

#### 4.3.1. First Round Delphi—Initial Idea Generation

The survey was administered using an online survey platform, primarily during a meeting attended by participants either in person or virtually. This setting facilitated real-time engagement and discussion among panelists. It was piloted by members of the research team. It took approximately 15 min to complete the survey. Responses to the survey were completely anonymized. The idea generation phase began with an open-ended question designed to elicit a wide range of perspectives on priority interventions in pediatric antimicrobial stewardship. The prompt was “Based on your experience and expertise, what are the 2–3 most critical interventions that should be prioritized to improve antimicrobial stewardship in pediatric healthcare systems?” Poll Everywhere platform enabled real-time data collection and convenient participation. Responses were anonymized to encourage candid feedback and reduce bias. Responses were analyzed using inductive thematic analysis. Three reviewers iteratively coded the data and collaboratively refined categories until consensus was achieved.

#### 4.3.2. Second Round Delphi—Prioritization

The thematically categorized interventions from Round 1 were displayed to participants, who were then reminded of the overall goal of the study: to prioritize areas for intervention in inappropriate antibiotic use in pediatric care. Some items were regrouped based on suggestions from participants, creating four broad thematic categories. Participants were asked to review the streamlined list closely and select the 3–4 interventions they believed were most important. To aid in achieving group consensus, participants were encouraged to consider the priority level of each intervention, weighing factors such as clinical impact, feasibility, and potential for reducing antibiotic misuse. This step allowed participants to reflect on the collective input from the first round while providing a structured approach to narrowing down the most critical areas for focus. Simple vote counts were used to analyze theme-related data. The items were then ranked based on the total number of selections, and items that received two votes or fewer were removed from the list. The process ensured that prioritization was grounded in both individual expertise and group consensus, setting the stage for the third round of refinement.

#### 4.3.3. Third Round of Delphi—Refinement

In Round 3 of the Delphi process, the reduced items from the second round were displayed to participants, highlighting the high-priority selections that had emerged. For two similar categories that received a significant number of suggested interventions, these high-priority items were consolidated into a single group, allowing participants to focus on the most critical areas. Participants were then asked to review these new group selections and make their final choices from that list, ensuring that the most impactful interventions were carried forward. Items that received two or fewer votes were dropped from further consideration. The final selections formed one new category and together with the remaining 2 categories, they were carried forward to Round 4 for final consensus building. This approach ensured that the most important interventions were thoroughly vetted and prioritized while also maintaining a balanced representation across all categories.

#### 4.3.4. Round 4—Final Consensus and Discussion

In Round 4, participants were presented with the outcomes of Round 3 to help direct their final discussions. The most important interventions were examined and discussed by experts across the following three categories: Care Settings, Prescriptions, and Strategies. Consensus was achieved through expert discussion, resulting in a focused set of stewardship priorities for pediatric antimicrobial stewardship. These discussions were transcribed in real time, and notes were reviewed to identify illustrative quotes that captured key themes, areas of consensus, and divergent perspectives. Quotes included in the Results section were selected to reflect the range and substance of expert input.

Figure 2 outlines a four-round method for reaching expert consensus. Round 1 focused on idea generation, followed by thematic analysis and initial voting in Round 2. Round 3 involved ranking and further prioritization, while Round 4 finalized priorities through deliberation and consensus-building.

## 5. Conclusions

This prioritization process identifies seven crucial areas for the advancement of ASPs. These areas were selected based on their potential for significant impact, existing gaps in implementation, and alignment with current challenges in antimicrobial use. The overlap among these domains emphasizes how antimicrobial stewardship interventions are interconnected and highlights the importance of coordinated efforts that address not only what is prescribed but also where and how antimicrobial stewardship is practiced. The study’s findings have meaningful implications for pediatric antimicrobial stewardship policies, though their adoption will depend on further validation and implementation efforts. The strong expert consensus on ICU and outpatient stewardship interventions suggests these areas should be prioritized in national guidelines, such as those from the Centers for Disease Control and Prevention or Infectious Diseases Society of America. The emphasis on shorter antibiotic durations and narrow-spectrum agents also supports updates to existing treatment guidelines for conditions like community-acquired pneumonia. By focusing on these priorities, ASPs can strengthen their capacity to improve appropriate antibiotic use, enhance patient safety, and combat AMR across healthcare settings.

## 6. Contributions to Literature

1. This study has the potential to significantly influence stewardship practices and enhance outcomes for children facing the increasing threat of antimicrobial resistance, as it is among the first to systematically prioritize pediatric ASP interventions through expert consensus.
2. The recommendations that follow will bridge the gap between the conceptual underpinnings of antimicrobial stewardship and their application in pediatric healthcare settings.
3. By establishing key priorities, this research lays the groundwork for future empirical studies, including interventional trials and implementation science research, to assess the effectiveness of identified ASP strategies.

## Figures and Tables

**Table 1:** Participant details.

Category	Participants (N/%)
**Role**	
Infectious Diseases Physician	10 (50%)
Pharmacist	8 (40%)
Other—Medication Safety Coordinator (Former ASP/ID)	1 (5%)
**Years of Experience**	
5–10	8 (40%)
11–20	10 (50%)
More than 20	1 (5%)
**Race**	
White	15 (75%)
Hispanic/Latin	2 (10%)
Asian	2 (10%)
**Gender**	
Male	9 (45%)
Female	10 (50%)
Total with demographic data	19 (95%)
Missing demographic data	1 (5%)

Percentages may not add up to 100 due to missing data.

**Table 2:** Participant Responses in Round 1.

Themes Generated	Responses from Participants (Priority Items)
1. Care Settings	Outpatient ASP Inpatient Care Unit ASP Urgent Care ASP Intensive Care Unit ASP
2. Prescriptions	Duration Narrow/Broad Spectrum for community-acquired infections Discharge antibiotics (IV to PO transition) Dose Pathogen-drug mismatches
3a. Clinical Practice Strategies	Novel methods of performing antimicrobial stewardship other than prospective audit with feedback Resource waste (environmental impact) Clinical pathway or practice guidelines development Improve consensus/goals of stewardship in infectious diseases as a whole Beta-lactam allergy de-labeling Antimicrobial stewardship in immunocompromised hosts Initiation of broad-spectrum antibiotics
3b. Programmatic and System-Level Strategies	Metrics Executive leadership involvement Diagnostic stewardship Patient care outcomes following Burnout amongst antimicrobial stewardship clinicians, especially among pharmacists Creation of more granular antibiotic measures that can help programs direct their activities to outlier services, units, diagnoses, and antibiotics Relationship building Antibiotic Harm Index De-escalation Continued surgical prophylaxis

**Table 3:** Delphi round 2 results.

Themes	Priority Items	Votes (N)	Consensus (%)
1. Care Settings	Outpatient ASP	13	65
	Inpatient Care Unit ASP	0	0
	Urgent Care ASP	2	10
	ICU ASP	6	30
2. Prescriptions	Duration	17	85
	Narrow/Broad Spectrum for community-acquired infections	5	25
	Discharge antibiotics (IV to PO transition)	1	0.5
	Dose	0	0
	Pathogen-drug mismatches	0	0
3a. Clinical Practice Strategies	Novel methods of stewardship other than prospective audit with feedback	2	10
	Resource waste (environmental impact)	2	10
	Clinical pathway or practice guidelines development	2	10
	Improve consensus/goals of stewardship to the ID division as a whole	2	10
	Beta-lactam allergy de-labeling	1	0.5
	Antimicrobial stewardship in immunocompromised hosts	1	0.5
	Initiation of broad-spectrum antibiotics	0	0
3b. Programmatic and System-Level Strategies	Metrics	10	50
	Executive leadership involvement	5	25
	Diagnostic stewardship	4	20
	Patient care outcomes following	3	15
	Burnout amongst antimicrobial stewardship clinicians, especially pharmacists	3	15
	More granular antibiotic measures that can help programs direct activities to outlier services, units, diagnoses, and antibiotics	3	15
	Relationship building	2	10
	Antibiotic harm index	1	0.5
	De-escalation	0	0
	Continued surgical prophylaxis	0	0

**Table 4:** Delphi Round 3 Results.

Theme	Priority Item	Votes (N)	Consensus (%)
Strategies	Metrics	13	65
	Patient care outcome tracking	2	10
	Diagnostic Stewardship	2	10
	Executive Leadership involvement	0	0
	Burnout amongst antimicrobial stewardship clinicians, especially among pharmacists.	0	0

**Table 5:** Themes, Priority Items and Supporting Quotes.

Theme	Priority Item/Subtheme	Number	Supporting Quote
Care Settings	ICU ASP	1.1	…so we have ICU stewardship up there, which I think is critically important due to the high level of clinical ambiguity in this setting. Patients are often complex, and stewardship decisions aren’t always straightforward. There’s rarely a single clear path. Yet the stakes are extremely high. For example, managing a multidrug-resistant organism in a patient on ECMO (extracorporeal membrane oxygenation) presents real challenges. This complexity makes the ICU a key area that deserves focused attention.
		1.2	Going back to another’s comment about the invisibility of harm to patients, which I completely agree with, I’d tie that back to the ICU. Our ICU physicians are typically on for seven days, so when a patient develops C. difficile two weeks later, they never see it. And I’d go even further: a lot of that harm is invisible to us as well. We often only recognize it when ID is consulted later, or the patient is started on another antibiotic. Then a stewardship pharmacist goes back, reviews the case history, and realizes, ‘Oh, they were on antibiotics for (a viral infection)—and that’s what led down this path.
	Outpatient ASP	1.3	Outpatient stewardship is critically important because it encompasses such a large and diverse portion of antibiotic prescribing in the U.S., from urgent care clinics to telehealth visits. It represents a substantial slice of overall antibiotic use and addressing it is essential. Many in this field are actively working on it, and it will remain a necessary area of focus. Outpatient stewardship requires approaches that account for the unique characteristics of prescribing in these settings
Prescriptions	Duration	1.4	Mean duration really matters partly because we rarely agree on what it should be. We know that shorter is generally better, and we have evidence that many conditions don’t need 10 or 14 days; some need 7, 5, or even just 3 days. But the problem is especially noticeable in the outpatient setting, where there’s a lack of clear guidelines, and urgent care providers may not be up to date with the latest studies. We’re still seeing community-acquired pneumonia treated for 10 or 14 days, often with the wrong antibiotic. Another issue comes at hospital discharge where sometimes a patient gets four days of antibiotics in the hospital, only needs one more, but gets sent home with a five-day prescription. So, what was intended as a five-day course unintentionally becomes 9 or 10 days. It’s a common and preventable problem.
		1.5	One of the appealing things about duration is that it’s a number and it’s concrete. It becomes the vessel that holds other antibiotic decisions, like whether to use a narrow- or broad-spectrum agent, or to go oral versus IV. Duration feels more measurable, more tangible. Thinking about it is feasible and desirable. It’s something I am motivated to work on.
	Narrow/Broad-Spectrum Antibiotics for Community- Acquired Infections	1.6	One of the challenges we face is demonstrating that longer antibiotic durations are actually harmful. That’s why I often refer to (a) study on adverse effects. They took the extra step of calling patients directly. It showed that those who received broad-spectrum antibiotics experienced more adverse effects, but even among patients who received narrow-spectrum, first-line therapy, about 25% still reported side effects. These might not have resulted in hospital visits, but they were distressing to the patient. That matters.
Strategies	Metrics	1.7	I think the biggest issue is actionable metrics. Working with DOT (days of therapy) per 1000 patient-days doesn’t tell us much, especially in aggregate. We need metrics that are more insightful and help guide program activities. I think about metrics in three categories: process measures, outcome measures, and - what we often overlook - program metrics. That third category is really about how our stewardship program is performing compared to others. We’ve worked on an impact scoring tool, and in trying to standardize it, we found that programs track their day-to-day interventions very differently, like whether they narrow, broaden, or stop therapy. So, I think standardizing how we track those interventions could really help us evaluate and improve stewardship across programs.
		1.8	I think we need to restructure the way we think about antibiotics. Starting a patient on vancomycin without a clear need—that’s a near miss. They could develop an acute kidney injury, and we don’t treat it like a safety issue, but we should. I see a lot of parallels between stewardship and patient safety work. If we start framing unnecessary antibiotic use as a safety event, something that could cause real harm, I think we’d see more engagement. From a metrics standpoint, I really like the idea of an antibiotic harm index. It’s marketable, and it helps shift the conversation. We need to talk more about antibiotics in terms of safety, that’s probably what will get people to pay attention.
	Patient Care Outcome Tracking	1.9	We have a lot of mixed data on the direct correlation between antibiotic exposure and resistance, or between exposure and adverse effects, especially when comparing five, seven, or ten-day courses. I think we need to dig deeper and focus on outcomes that truly matter to patients. For example, a study might show no difference in the incidence of diarrhea between five and ten days, but what about the number of days a patient had diarrhea? That often isn’t assessed. So, I would love to see just more of us do a little better job of trying to make a compelling argument that actually matters to the patients getting the antibiotics
	Diagnostic Stewardship	2.0	Before working in ID, I was in the ICU, and it’s interesting. While we often talk about ICU teams struggling with antibiotic use, they also have concerns about how we in ID order diagnostic tests. I think there’s a real opportunity for us to steward our own diagnostic practices, being more thoughtful about the cost of care and asking what we’ll actually do with the results. This is an area where we might have more control compared to others. There are certain tests, like some syndromic panels, that are being overused, and I’ve seen firsthand how quickly things can spiral. We need to set an example, because when we’re not careful with our own diagnostic decisions, it can influence and even amplify misuse by others.

## Data Availability

Data is available from the corresponding author upon request.

## Abbreviations

The following abbreviations are used in this manuscript:

AMR	Antimicrobial Resistance
ASP	Antimicrobial Stewardship Program
ID	Infectious Disease
ICU	Intensive Care Unit
CAP	Community-Acquired Pneumonia
OPerAtiC	Optimizing PERioperative AnTibiotICs
DOT	Days of Therapy
ECMO	Extracorporeal Membrane Oxygenation
